# Differences in Physiological and Biochemical Attributes of Wheat in Response to Single and Combined Salicylic Acid and Biochar Subjected to Limited Water Irrigation in Saline Sodic Soil

**DOI:** 10.3390/plants9101346

**Published:** 2020-10-12

**Authors:** Emad M. Hafez, Ahmed M. S. Kheir, Shimaa A. Badawy, Emadeldeen Rashwan, Mohamed Farig, Hany S. Osman

**Affiliations:** 1 Department of Agronomy, Faculty of Agriculture, Kafrelsheikh University, 33516 Kafr El-Sheikh, Egypt; shamsbadawy2000@yahoo.com; 2 Agricultural Research Centre, Soils, Water and Environment Research Institute, 12112 Giza, Egypt; ahmed.kheir@arc.sci.eg; 3 Agronomy Department, Faculty of Agriculture, Tanta University, 31111 Tanta, Egypt; emad.rashwan@agr.tanta.edu.eg; 4 National Water Research Center, Water Management Research Institute, Delta Barrage, P. O. Box 13621/5 Qalubia, Egypt; freegragb@gmail.com; 5 Faculty of Agriculture, Tottori University, 4-101, Koyamacho-minami, Tottori 680-8550, Japan; 6 Department of Agricultural Botany, Faculty of Agriculture, Ain Shams University, 11566 Cairo, Egypt; hany_osman1@agr.asu.edu.eg

**Keywords:** wheat, depletion of available soil moisture, biochar, salicylic acid, antioxidant enzymes, nutrient uptake

## Abstract

Given the expectancy of the water supply becoming scarce in the future and more expensive, water conservation during wheat production processes has become very crucial especially in saline sodic soil. Biochar and salicylic acid (SA) were used to assess the potential to alleviate the influences of depletion of available soil moisture (DAM) on physicochemical, physiological, biochemical attributes, as well as wheat production absorption (*Triticum aestivum* L. cv. Misr 1) and macro-elements. Two seasons (2018/2019 and 2019/2020) of field trials were investigated using twelve combinations of three water treatments (50%, 70%, and 90% DAM) and foliar- and soil-applied treatments (control, biochar, salicylic acid, and biochar + SA). Biochar treated plots amplified soil physicochemical attributes, leading to improved physiological traits and antioxidant enzymes, as well as yield related traits under water limitation conditions in both years. Similarly, synergistic use of biochar and salicylic acid greatly augmented the designed characteristics such as chlorophyll a, b, K^+^ content, relative water content (RWC), stomatal conductance, photosynthetic rate, and intrinsic water use efficiency, whilst exhibited inhibitory effects on proline content, electrolyte leakage, Na^+^ content SOD, POX, CAT, and MDA, consequently increased 1000-grain weight, number of grains spike^−1^, grain yield, as well nutrient uptake (N, P, K) under water limitation condition in both years, followed by treatment of sole biochar or SA compared to unamended plots treatment (control). Wheat productivity achieved further increasing at 70% DAM alongside synergistic use of biochar and SA which was on par with 50% DAM under unamended plots (control). It is concluded from the findings that coupled application of biochar alongside salicylic acid accomplished an efficient approach to mitigate the injurious influences of water limitation, along with further improvement of the soil, physiology, biochemical attributes, and wheat yield, as well nutrient uptake, under saline sodic soil.

## 1. Introduction

Wheat (*Triticum aestivum* L.) is worldwide the most important food and feed cereals with productivity superior to that of rice and maize. It is greatly cultivated in semiarid zones [1], and currently, cultivation is prolonged to arid zones, which possibly assists to undertake the future food security in problematic zones where water stress and soil salinity are the greater troubles in declining wheat crop [2]. Wheat accounts for 40% of the world edible dry and equals to 70% of daily calories consumption in numerous developing countries [3]. Thus, augmenting wheat production is the pivotal national aim to seal the gap between production and intake, particularly in developing terrains [4]. Yearly, the intake of wheat in Egypt is roughly 18.9 Mt, while the Egyptian production is roughly 9.0 Mt [5,6]. Wheat grain yield is a polygenic characteristic and is likewise affected by a number of environmental factors, including water stresses [7] and soil salinity. Thus, it is instantly extremely essential to develop new management practices to cope with this imminent dilemma of food security with low usage of water and soil degradation. 

Plants are unceasingly subjected to a broad set of ecological stressors, such as water stress, soil salinity, cooling, and heating, resulting in impairment to crop production by more than 50% [8,9]. Water stress is the highly imperative abiotic stress for crop reduction under arid and semiarid zones and, ultimately, a menace regarding universal food security in the upcoming era [10]. Water stress has been found harmful to the formation of seedlings and to the growth and physiological attributes of different crops such as wheat [11,12]. Wheat plants subjected to water stress lessens the metabolic activity, reduces its biomass accumulation, and diminishes photosynthesis activity by curtailing chlorophyll content in leaves, leading to crop reduction [13]. Water shortage is able to prolong vegetative growth stage and decrease the leaf area, reflecting negatively on crop production [14]. Furthermore, increasing leaf temperature and reducing leaf water potential, turgor potential, and stomatal movements owing to crops’ exposure to water deficit, which eventually hinder plant growth [15]. Water stress hinders physiological and metabolic processes in leaves and induces oxidative stress by increasing generation of reactive oxygen species (ROS) [8,16]. Crops may survive under abiotic stress conditions across different mechanisms that permit them to amend water transport in response to water stress.

Plants produce important signaling molecules which increase their tolerance to ecological incentives and are capable of declining injurious impacts. Including these signaling compounds, salicylic acid (SA) as a phytohormone plays a multifaceted role in plants to increase tolerance of abiotic stressors including drought and salinity [17,18]. Furthermore, SA positively influences plant development stages, from seed emergence to final crop [19]. Foliar spraying of salicylic acid has been exhibited to increment drought and salinity tolerance by regulating photosynthesis, stomata opening, cell growth and reduce the ion leakage as well as the injury owing to oxidative stress [20,21,22]. SA has been well-known to play an intriguing role in increasing the relative water content in leaves [23] and osmotic regulation [24] in plants subjected to stressors.

The injurious impacts of water stress could be reconciled by the application of nutrients to the soil, leading to improvement of water stress tolerance [25]. Biochar is one such amendment that could diminish the deleterious effects of water stress in the plant development stage [26]. Soil amendment as biochar is confirmed to alleviate adverse impacts of water stress and soil salinity stressors on crops [27]. Biochar is the carbon-rich product of thermally cracked biomass feedstock in an oxygen limited environment [28]. Furthermore, biochar includes considerable amounts of elements like Ca^2+^ and Mg^2+^ and inorganic carbonates, which is valuable for plant development. Biochar can enhance soil health, soil permeability, and carbon sequestration [29]. It is stated that biochar could improve productivity by stimulating microbe activity in the rhizosphere and enhancing soil water holding capacity [30]. Furthermore, biochar greatly augmented soil surface area owing to its highly porous structure, leading to improved cation exchange capacity (CEC) [31]. Therefore, abundant amounts of nutrients could be preserved in soil, boosting nutrient uptake, resulting in better crop production [32]. In our prior report, we attained that biochar has the capability to increment soil moisture content primarily due to its high porosity [33]. Besides, it was observed that biochar could have the capability to decline the harmful effects of soil salinity and proposed three mechanisms i.e., (1) transient binding of Na^+^ on its exchange site, and therefore declining Na^+^ absorption; (2) augmenting K^+^ content in soil solution, and thus sustaining Na^+^/K^+^ ionic equilibrium to lessen Na^+^ absorption; (3) increment of soil moisture content could trigger dilution influence which eventually can lead to decrement in Na^+^ absorption [34]. In addition, biochar has the capability to adsorb high salt, therefore, could lessen Na+ absorption in plants and relieve the injury effect triggered by soil salinity [35]. Biochar has become a research hotspot in the field of agricultural practices recently, particularly for sustained development of soil quality [36].

Water limitation alongside soil salinity is the most widespread co-occurring abiotic stressor and menace of universal food security, particularly in arid and semi-arid zones. Therefore, it is immediately required to present integrated approaches such as application of soil amendments as biochar and exogenous application as salicylic acid. The overall goal of this research was to achieve the integrated use of salicylic acid and biochar on the soil physicochemical properties, physiological, biochemical attributes and the productivity of wheat, as well as nutrient uptake under water limitation conditions in saline sodic soil.

## 2. Results

This experiment was performed to assess the coupled impact of salicylic acid as foliar spraying and biochar as soil amendment on soil, physiology, biochemical, and nutrient uptake, as well as yield and related traits of wheat plants under deficit irrigations in saline sodic soil. The impact of salicylic acid and biochar and their combined use displayed the varying outcomes when exposed to analysis. The field experiment for 150 days presented the following results during the growing seasons 2018/2019 and 2019/2020. 

### 2.1. Soil Physicochemical Parameters

The impact of salicylic acid and biochar on soil chemical parameters of wheat after harvesting under different deficit irrigations (50%, 70%, and 90% depletion of available soil moisture (DAM)) in saline sodic soil are given in (Table 1). In order to detect the after impacts of added treatments on soil, the soil was collected and analyzed for pH, EC, ESP, Na^+^, K^+^, Ca^2+^, and Mg^2+^ after harvesting wheat plants from each treatment (Table 1), Ca^2+^, Mg^2+^ treated. The application of biochar alone significantly decreased the pH, EC, ESP, and Na^+^, while significantly increased K^+^, Ca^2+^, and Mg^2+^ of the soil compared to application of sole salicylic acid followed by control treatment. The combined application of salicylic acid and biochar gave the maximum decrease in the pH, EC, ESP, and Na^+^ and the maximum increase in K^+^, Ca^2+^, and Mg^2+^ of the soil in comparison with the application of salicylic acid and biochar alone and with that of the control under 50% depletion of available soil moisture, followed by 70% and 90% DAM in saline sodic soil. Under mid and severe water deficit irrigation, i.e., 70% and 90% DAM, respectively, the combined application of salicylic acid and biochar significantly improved soil physicochemical properties compared to the application of salicylic acid and biochar alone and with that of the control. The minimum values of soil physicochemical properties were conserved in respective control treatment plots. Besides, highly significant correlation was perceived between post-harvest soil parameters and yield attributes as well nutrient uptake.

### 2.2. The Percent of Na^+^ and K^+^ Content in Leaves

Statistical analysis showed that the wheat plants treated with salicylic acid, biochar, and mix (both SA and biochar) had significantly (*P* < 0.05) lower Na^+^ and higher K^+^ as compared to the control. The synergistic application of SA and biochar led to the maximum increase in K^+^ and the minimum decrease in Na^+^ under normal conditions, i.e., 50% DAM compared to severe deficit irrigation (90% DAM). However, this impact was minimized by the addition of salicylic acid and biochar singular or combined. Limited moisture availability i.e., 70% DAM when combined with SA and biochar considerably improved K^+^ of wheat leaves and decrease Na^+^ (Table 2) which was significantly on par with wheat plants irrigated at 50% DAM with the untreated plants in both years. Likewise, combined with SA and biochar considerably improved K^+^ of wheat leaves and decreased Na^+^ (Table 2) which was significantly on par with wheat plants irrigated at 70% DAM with the untreated plants in both years.

### 2.3. Physiological Properties of Wheat Plants

It was attained that control plants under each set of water deficit irrigation had the lowest chlorophyll a, b and relative water content, whilst they had the highest proline content and electrolyte leakage as shown in (Table 3). An increment in chl a, b, and relative water content (RWC) was detected with singular or coupled application of salicylic acid and biochar, while a decrement in proline content and electrolyte leakage was observed with singular or coupled application of salicylic acid and biochar. The maximum chl a, b, and RWC and the minimum proline content and electrolyte leakage were perceived in the plants treated with combined application of salicylic acid and biochar under 50% DAM conditions, followed by 70% and 90% DAM. The maximum chl a (1.68 and 1.63 mg g^−1^ FW), ch b (0.76 and 0.93 mg g^−1^ FW), and RWC (94.98% and 91.66%), respectively in 2018/2019 and 2019/2020, were observed under 50% FC conditions when combined with the mix of SA and biochar, which were more compared with its respective control. The minimum proline content (7.17 and 6.14 µ mol g^−1^ FW) and electrolyte leakage (13.47% and 14.98%), respectively in 2018/2019 and 2019/2020, were observed under 50% FC conditions when combined with the mix of SA and biochar which were more compared with its respective control. It was observed that plants treated with 70% DAM and combined SA and biochar considerably declined proline content and electrolyte leakage of wheat more that wheat plants irrigated at 50% DAM with the untreated plants in both years (Table 3).

**Figure 1:**
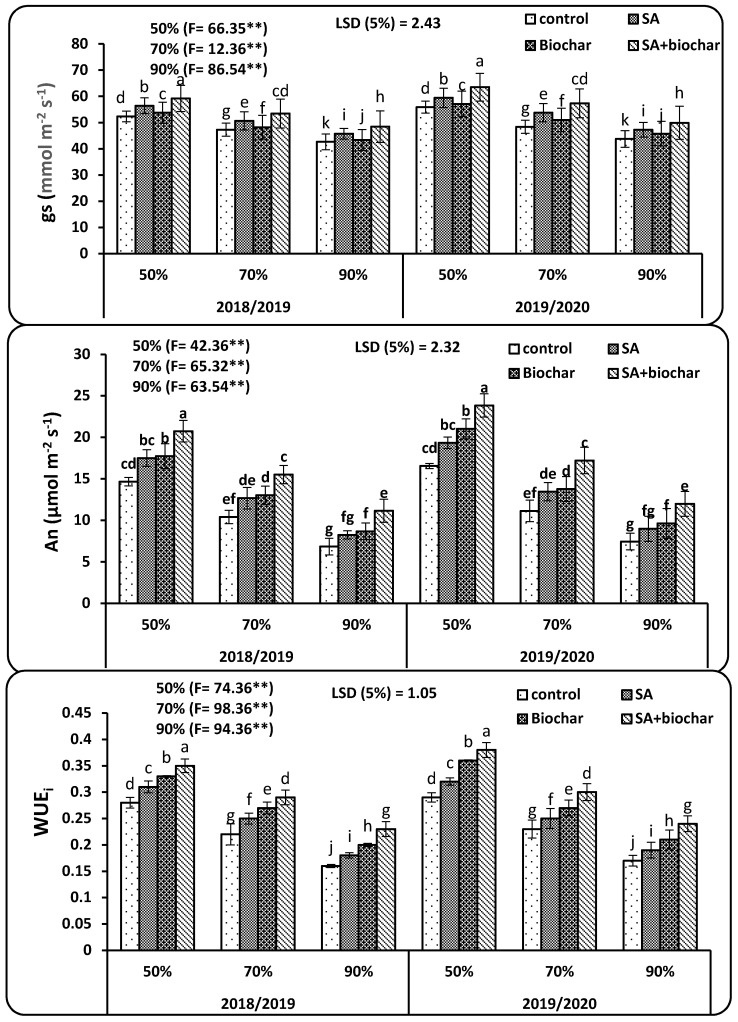
Stomatal conductance (g_s_), leaf photosynthesis (An), and intrinsic water use efficiency (WUE_i_) of wheat leaves as treated by (control, salicylic acid, biochar, and SA + biochar) under different three irrigation conditions (50%, 70%, and 90% DAM) in saline sodic soil. Error bars indicate ± SE (*n* = 3). SE indicates standard error. Mean values designed by the same letter in each column are not significant according to Tukey’s multiple range test. ** denote significance at *P* < 0.01.

The influence of SA, biochar, and their combination on stomatal conductance (g_s_), leaf photosynthetic rate (An), and intrinsic water use efficiency (WUE_i_) were exposed in (Figure 1). Regardless of SA and biochar application, reduced irrigation (70% and 90% DAM) greatly declined both An and g_s_ compared to 50% DAM. Singular application of SA and biochar achieved positive impacts on both An and g_s_ in all different irrigation conditions, especially in 70% and 90% DAM under saline sodic soil compared to control (untreated plots). There was a higher remarkable significant effect for the coupled application of SA and biochar on g_s_ and An in both years of study, resulting in maximum WUE_i_. Maximum increment in g_s_, An, and WUE_i_ were observed under stress condition in response to SA, biochar, and their combined application as compared with stressed plants (control plots). Limited moisture availability i.e., 70% DAM when combined with SA and biochar considerably gave the best findings for g_s_, An, and WUE_i_ of wheat (Figure 1), which was significantly on par with wheat plants irrigated at 50% DAM with the untreated plants in both years. Likewise, the same trend was found for treatment of 90% DAM when combined with SA and biochar considerably gave the best findings for g_s_, An, and WUE_i_ of wheat, which was significantly on par with wheat plants irrigated at 70% DAM with the untreated plants in both years.

### 2.4. The Antioxidant Enzymatic Activity

**Figure 2:**
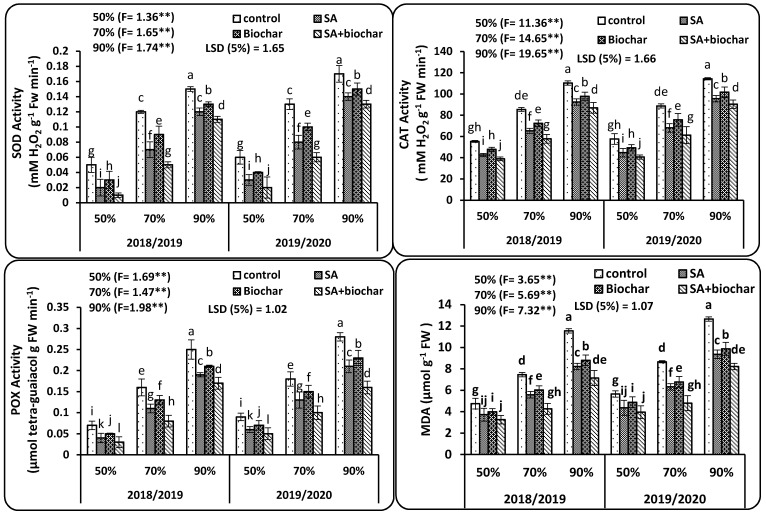
Superoxide dismutase (SOD), catalase (CAT), peroxidase (POX), and lipid peroxidation (MDA) of wheat leaves as treated by (control, salicylic acid, biochar, and SA + biochar) under different three irrigation conditions (50%, 70%, and 90% DAM) in saline sodic soil. Error bars indicate ±SE (*n* = 3). SE indicates standard error. Mean values designed by the same letter in each column are not significant according to Tukey’s multiple range test.

Deficit irrigation conditions i.e., (70% and 90% DAM) significantly augmented the antioxidant enzymatic activity of wheat leaves (Figure 2) i.e., activity of SOD, CAT, POX, and MDA in both years of study. The attained findings in Figure 2 presented that activity of SOD, CAT, POX, and MDA significantly stimulated in the stressed plants in both years. The maximum increases in activity of SOD, CAT, POX, and MDA were obtained in the plants that neither treated by SA nor biochar under severe deficit irrigation i.e., 90% DAM compared to plants treated by normal irrigation i.e., 50% DAM. Inversely, SOD, CAT, POX, and MDA were noticeably affected by SA or biochar addition under deficit irrigation conditions i.e., 70% and 90% DAM. Likewise, addition of singular SA and biochar or their combination resulted in improved physiological attributes which led to decrement in activity of SOD, CAT, POX, and MDA. Under deficit water irrigation conditions, the plants activate the defense system to scavenge reactive oxygen species which cause oxidative stress to plant organelles. In this study, wheat plants subjected to deficit irrigation displayed significant increases in SOD, CAT, POX, and MDA activities mainly in the plants that received 90% DAM followed by the plants that received 70% DAM in both years (Figure 2). Combined application of SA exogenously sprayed and biochar to soil under treatment of 70% DAM resulted in an improved activity of antioxidant enzymes around the optimum level like the control plants at 50% DAM. The greatest outcomes were found with the plants treated with coupled treatment and that received 70% DAM in both years with was on par with 50% DAM with control plots (untreated plants).

#### Yield Related Traits and Productivity

Single salicylic acid and biochar as well as their combination resulted in significant increase in number of grains spike^−1^, 1000 grain weight, as well as grain yields as recorded in (Table 4). As shown in Table 4, number of grains spike^−1^, 1000 grain weight as well as grain yields were severely suppressed under severe deficit irrigation i.e., 90% DAM treatment in saline sodic soil. The application of salicylic acid and biochar alone or combined significantly improved the number of grains spike^−1^, 1000 grain weight, as well as grain yields in comparison with control. The maximum number of grains spike^−1^, 1000 grain weight, as well as grain yields was recorded in the treated plant which was irrigated at 50% DAM, followed by 70% and 90% DAM. The maximum number of grains spike^−1^, 1000 grain weight, as well as grain yields was perceived in the plants treated with combined application of salicylic acid and biochar under 50% DAM conditions, followed by 70% and 90% DAM. The maximum number of grains spike^−1^ (57.96 and 56.74), 1000 grain weight (56.44 and 57.98 g), and grain yield (6.67 and 6.23 t ha^−1^), respectively in 2018/2019 and 2019/2020, were observed under 50% FC conditions when combined with the mix of SA and biochar which were more compared with its respective control. It was observed that plants treated with 70% DAM and combined SA and biochar considerably increased yield and related traits of wheat more that wheat plants irrigated at 50% DAM with the untreated plants in both years (Table 4).

### 2.5. Nutrient Uptake

Regarding the nutrient uptake of the grains, nitrogen, phosphorus and potassium uptake was significantly increased with the alone/combined application of salicylic acid and biochar. The maximum increment was noted with the coupled application of salicylic acid and biochar, followed by biochar and salicylic acid under all DAM irrigation conditions (50%, 70%, and 90% DAM) in comparison to control treatment plots. The minimum increment was found in control treatment plots. The response of N, P, and K uptake in grains to the stresses varied with foliar and soil treatments (Table 4). In the combined treatment (SA and biochar), uptake of N, P, and K dramatically increased under water deficit and saline sodic soil, while lower N, P, K uptake were detected in control (untreated plants). Data regarding N, P, and K uptake showed that the application of SA and biochar considerably improved the nutrient uptake of wheat plants under different deficit irrigations (Table 4). The maximum N, P, and K uptake were recorded at 50% DAM in comparison with other DAM conditions and their respective control. The maximum N uptake (103.85 and 111.03 kg ha^−1^), P uptake (62.85 and 62.36 kg ha^−1^), and K uptake (163.74 and 174.36 kg ha^−1^), respectively in 2018/2019 and 2019/2020, were observed under 50% FC conditions when combined with the mix of SA and biochar which were more compared with its respective control. It was observed that plants treated with 70% DAM and combined SA and biochar considerably increased yield and related traits of wheat more that wheat plants irrigated at 50% DAM with the untreated plants in both years (Table 4).

## 3. Discussion

Environmental stressors i.e., water deficit and soil salinity have detrimental influences on plant growth, development, and metabolism during the whole crop life cycle. So, there is a dire requirement to attain practical and effective approaches to retain adequate soil moisture, water uptake, and ion balance for crops under water deficit and saline soil. Various techniques, which are cost effective like exogenous spraying with salicylic acid, biochar application to soil, and planting of salt-tolerant crops like wheat, have been used to deal with water deficit and soil salinity. So, their combinative impact on water deficit and soil salinity requires to be evaluated. This experiment sought to illustrate the defensive role of salicylic acid and biochar and its combined application in alleviating the water deficit through improving soil physicochemical, the plant physiological, biochemical attributes, nutrient uptake, as well as yield and related traits of wheat plants under 50, 70, and 90% depletion of the available soil moisture (DAM) in saline sodic soil during 2018/2019 and 2019/2020. Our findings manifested that combined application of exogenously salicylic acid and biochar to soil has been more effective to increment yield under water deficit conditions in saline sodic soil.

It was stated that SA plays major roles in regulating plant growth, development, and the responses to environmental stresses. This agrees with the finding of [37]. Likewise, given that biochar plays a major role in decreased nutrient leaching, better soil physicochemical attributes in saline soils [38]; improves CEC by improving soil health. Application of biochar to the soil is considered a practical approach to decrease soil moisture content because of its properties to get better soil hydrological parameters [39], which in turn enrich plant physiological and biochemical responses. It was of merit to perceive that biochar and SA coupled application had the uppermost intrinsic water use efficiency (WUEi) under water limiting condition in saline sodic soil [40]. The impact of biochar to avoid water deficit and soil salinity has been well documented. It was noted that biochar could have a superior capacity to quickly adsorb Na+ on its exchange site, leading to dropping Na+ balance content in soil solution. Moreover, higher water holding capacity of biochar can trigger dilution of salt and thus decrease osmotic stress [41]. In addition, biochar can augment inorganic content in soil solution by releasing mainly K^+^, Ca^2+^, and Mg^2+^, resulting in declining Na^+^ absorption in crops. In the present experiment, it was observed that coupled application of biochar and SA had a great impact in mitigating water deficit conditions alongside saline sodic soil [42].

Physiological properties are imperative attributes and play a pivotal function in crop development and productivity. In this experiment, severe deficit water irrigation conditions i.e., 90% DAM led to a considerable lessening of chlorophyll a, chlorophyll b, RWC, gs, and photosynthetic rate, as well WUEi in wheat plants (Table 2, Figure 1) in saline sodic soil. It was observed that deficit water led to an adverse impact on photosynthesis by hurting the ultrastructure of chloroplasts and inhibiting the synthesis of vital pigments, inhibiting the Calvin cycle and the electron transport chain, and stimulating a carbon dioxide lack by closing the stomatal pores in saline sodic soil [43,44]. The decrease photosynthetic pigments under insufficient irrigation can be owing to accumulation of proline content, MDA, and electrolyte leakage (Table 2, Figure 2) in saline sodic soil. Besides, photosynthesis suppression could be attributed to the deficit irrigation alongside with saline sodic soil, thus hindering the nutrient uptake (Table 3) [45,46]. Water deficit declined the photosynthetic rate and stomatal conductance, resulting in lesser water absorption due to depletion of water availability. It was found that addition of biochar to soil increased water holding capacity (WHC) as well as physicochemical properties [47], thereby increasing the available soil water, resulting in a higher photosynthetic rate [48]. Notably, the applied biochar to the soil modified the soil pH. The acidic soil improves nutrient retention and availability in the root zone [49]. In addition, the coupled use of biochar and SA led to positively increment in wheat crop as well as plant nutrient uptake when compared to their singular addition and untreated plots. Moreover, exogenous addition of salicylic acid ameliorates water deficit [50], increases photochemical efficacy as well as the enzymes activity [51], leading to better physiological attributes under coupled addition of biochar and salicylic acid [52]. 

The singular exogenous spraying with salicylic acid and application of biochar to the soil and their synergistic use under deficit water irrigation conditions ameliorated soil properties and physiological traits, resulting in improving yield and its related traits by alleviating the harmful impacts of water deficit and saline sodic soil. Applied SA was observed to regulate soluble and nutrient absorption under water deficit conditions [24,53]. In addition, salicylic acid could stimulate cell division, leading to the stimulation of growth development and antioxidant activity [18]. The enhancement of growth development, physiological attributes, photosynthetic performance, and biochemical activity under the addition of foliar spraying SA was linked to high crop productivity (Table 3, Figure 2). Furthermore, the enriched wheat yield following upon biochar addition in 2018/2019 and 2019/2020 can be ascribed to an amendment in the soil physical and chemical attributes. Enhanced soil physical attributes will clearly impact the soil–water interactions and thus stimulate a further efficient absorption of water from the root zone [35]. The increase in yield can be attributed to the delayed senescence of plant organs and following prevention of the premature loss of an anthesis and grains. Furthermore, SA likewise stimulates cell division and cell enlargement [42], resulting in an increment to the yield related traits. SA can prompt an increment in the total phenolic concentration of plants which counteract auxin degradation, thus improving grain yield [50]. Additionally, [53] stated that SA application augmented the number of grains in spikes, directing the flow of metabolites to the developing grains, which improved the 1000-grain weight and, thus, the grain yield at harvest in plants under water deficit. 

It was obvious from our findings that the exogenous spraying with salicylic acid or application of biochar to soil and their integration improved the physiological attributes under both water deficit and non-water deficit. This could be owing to the defensive impact of SA, which may boost photosynthesis and the nutrient uptake under deficit irrigation in saline sodic soil [37]. Singular biochar treatment could decrease the soil bulk density and increase soil surface area owing to its porous structure, leading to increasing the capability to uptake and hold water [38]. Furthermore, [40] affirmed that biochar possibly increases soil aggregate stability and therefore increases soil water holding under water deficit. Likewise, [39] demonstrated that the biochar porous structure declined evapotranspiration and augmented aeration and soil water retention as well as diluted ions concentration under saline soil, thus stimulate an appropriate soil for crop development. Furthermore, salicylic acid plays a pivotal role in declining chlorophyll degradation and the increase of lipid peroxidation and electrolyte leakage under severe deficit irrigation [44]. SA helps to restore the stomata pore opening, thereby augmented stomatal conductance and photosynthetic rate, leading to increased WUEi [23]. Razmi et al. [24] demonstrated that spraying SA could increase the K content and decrease Na in the membranes of guard cells (Table 2), which are imperative for increasing stomatal conductance, and improving chlorophyll content. In our study, it was found an increase in lipid peroxidation and electrolyte leakage under water deficit conditions (Figure 2 and Table 3), leading to the breakdown of plant cells. The foliar spraying of SA was declined oxidative stress by increased antioxidant enzyme activity which impeded ROS overproduction [21]. Based on that, exogenous application by salicylic acid decreased MDA and EL.

The enzymes activity is a well-known protective system versus reactive oxygen species. Moreover, extreme reactive oxygen species flow could assist to upset the antioxidant protective system, which is the major impact of water deficit. In our experiment, the activity of antioxidant enzymes, such as SOD, CAT, and POX, boosted when wheat plants were subjected to water deficit (Figure 2). The augmented enzymes activity resulted in the tolerance of wheat plants to water deficit and saline soil by modifying the virtual amounts of reactive oxygen species [23]. Superoxide dismutase activity delivers an initial-line protection system versus reactive oxygen species in harmony with POX and CAT. Water deficit conditions markedly augmented POX activity compared to untreated plots and was observed to be liable for the detoxification of H_2_O_2_ to H_2_O and O_2_ [8]. Furthermore, it was observed that the synergistic use of salicylic acid alongside biochar further improved the activity of these enzymes under water deficit in our study. Application of SA and biochar declined the harmful impact of water deficit and saline soil, restoring plant development and physiological attributes, and activating enzymes (catalase, peroxidase, and superoxide dismutase) due to salicylic acid playing an important role in transcription and/or translation resulting in inhibition of oxidative stress [46]. SA could also play an important role as a signaling molecule for the regulation of phytochelatin biosynthesis pathway activities [18].

Water deficit i.e., 70% and 90% DAM, considerably declined the uptake of N, P, and K, in wheat grains compared with well watered treatment i.e., 50% DAM (Table 4). The foliar spraying with SA and application of biochar to soil singular or combination appreciably augmented N, P, and K uptake in grains of wheat plants under water deficit conditions in saline sodic soil (Table 4). This was because the roots were the first organs to encounter the water and salt stress [13,52]. Ion absorption was greatly substantially affected by soil moisture treatments. The findings might be ascribed to the decrement of the nutrient in wheat plants as soil moisture declined, which can be owing to decreasing the solubility of elements in the soil where the films are thin and the path length of movement boosts; thus, drive of cations and anions to root is declined. Besides, high tension exerts a physiological impact on root, elongation, turgidity, and number of root hairs decline with increment of tension [7]. Water and salt stress greatly declined ion nutrient absorption in terms of N, P, and K, strengthening results from previous experiments that nutrient status is sensitive to the previous stresses. Increment of the depletion of available soil moisture from 50 to 90% declined nutrient absorption of wheat grains. Water availability in association with ion absorption exposed that nutrient absorption i.e., N, P, and K will decline without adequate soil water available to the plant. It can be ascribed to a declined transpiration rate to transport nutrients from roots to leaves [4]. It was observed that application of 50% DAM has been advocated to further effectively improve nutrient absorption. When wheat plants are exposed to a water deficit i.e., 70% and 90% DAM, the effectiveness of salicylic acid and biochar solely or coupled are higher than the control treatment (neither SA nor biochar). The maximum nutrient absorption N, P, K in the grains of wheat could be ascribed to the physiological mechanisms engaged in osmoregulation. SA prompted N, P, and K absorption in wheat grains as well declined Na content in the leaves. Individual treatment of biochar might alleviate the harmful impact of water deficit on wheat yield by improving water holding and distribution. It might however be observed that the co-treatment with SA further enhanced the performance under this water limiting condition. In water deficit prone condition, available soil water is crucial for nutrient uptake and holding in the root zone, mainly, the essential nutrients; nitrogen (N), potassium (K), and phosphorus (P) [11,22]. Consequently, biochar plays a pivotal role as an absorber of nitrogen, thus decreasing N leakage [4], and enhances its availability under water deficit condition [54,55]. Furthermore, the sole application of biochar increases the soil organic matter, which enhances the hydrologic buffer potential as well as WHC [18]. This finding delivers more elucidation for the enhanced soil water holding with biochar applied to soil which also improves soil structure [25,27].

Increased DAM hindered yield-related attributes i.e., numbers of grains spike^−1^ and 1000-grain weight, which is in harmony with [26]. The impact of foliar spraying with SA was linked to an increment in numbers of grains spike^−1^ and 1000-grain weight (Table 4). Similar results for foliar spraying SA have been stated for wheat by [28] which relieved the negative impacts of water deficit on yield related-traits in wheat [30]. This could be ascribed to an increase of nitrogen absorption, metabolic managed, and thereby increment grain *yield* when salicylic acid was added [34]. Moreover, it has been confirmed that SA has a central impact in growing canopy photosynthesis and metabolic transport of photosynthetic assimilates to wheat grains through the influence on phloem loading [35]. Application of SA might relieve the oxidative damage of wheat through transcriptional regulation of multiple protective pathways and improving reactive species biosynthesis [36]. 

## 4. Materials and Methods 

### 4.1. Study Site

A field experiment was implemented from December to April in 2018/2019 and 2019/2020 at the Sakha Agricultural Research Station (SARS) Farm, Kafr El-Sheikh, Egypt (Latitude: 31°6’ N/ Longitude: 30°56’ E) to assess the effect of exogenous application with salicylic acid and soil application with biochar alone and in combination on the soil, physiological and biochemical characteristics, as well as yield and nutrient uptake of wheat under water stress in saline sodic soil. 

The climate conditions in the two growing seasons were set as follows in Table 5.

### 4.2. Experimental Design and Crop Management

The experiment was laid out in split-plot arranged into randomized complete blocks consisting of three different water management modes (50%, 70%, and 90% depletion of the available soil moisture) allocated in main plots combined with untreated plots (control), sole salicylic acid, sole biochar, and combined (salicylic acid + biochar) were allocated in sub plots with three replicates. The sub-plot size was 12 m^2^ (3 × 4). The rows were 4 m long and spaced 15 cm apart. There were 2 m gaps between the blocks and 1.5 m alleys between the main-plots to avoid lateral water movement and other interferences. Healthy and uniform wheat grains (Misr 1 cultivar) were attained from the Wheat Research Center, Sakha, Kafr El-Sheikh, Egypt and were planted at a seeding rate of 142.8 kg ha^−1^. The grains were surface sterilized with 1% (v/v) NaClO for 5 min and then thoroughly washed several times with double-distilled water. The seeds were left to air dry for 1 h and then prepared for sowing. The previous crop was maize (*Zea mays* L.) in both seasons. 

Soil was ploughed twice, ridged, and divided into plots. Phosphorus fertilizer was applied during the soil preparation at the rate of 35 kg P_2_O_5_ ha^−1^ in the form of super phosphate 15% P_2_O_5_. Potassium fertilizer was applied as one dose directly before the first irrigation at the rate of 57 kg K_2_O ha^−1^ in the form of potassium sulfate 48% K_2_O. Nitrogen fertilizer was applied at two equal doses directly before the first and second irrigations at a rate of 180 kg N ha^−1^ in the form of urea 46.5% N. Other agronomic practices such as protecting wheat plants from weeds and diseases were completed in a timely manner.

During seed preparation, soil samples were collected at 0–30 cm depth using an auger to determine the physicochemical attributes of the experimental soil. Soil samples were air-dried and passed through a 2-mm sieve for physicochemical properties analysis (Table 6).

The initial soil physicochemical analysis in the two growing seasons was set as follows in Table 6.

Soil moisture constants before planting in the two growing seasons 2018/2019 and 2019/2020 are shown in Table 7.

Available soil water (ASW) was calculated based on the following equation:

ASW = (FC − PWP) × Bd × V

where FC and PWP are the gravimetric soil–water content (%) at FC and PWP, respectively, Bd refers to the value of soil bulk density (g cm^−3^) and V indicates the soil layer volume (m^3^) at the depth of the root zone.

### 4.3. Preparation of Biochar and Salicylic Acid

Foliar salicylic acid (hydroxybenzoic acid-2, C_6_H_4_(OH)COOH) was sprayed twice at a rate of 200 mg L^−1^ (192 g ha^−1^) at 50 and 70 days after planting. 

Biochar applied in this current research was equipped during slow pyrolysis of rice husk and corn stalk (1:1) at 350 °C under anaerobic circumstances with a mean residence time of 3 h [31]. The chemical composition of biochar is: pH (1:5 biochar:water extract) 7.60 ± 0.02; EC (1:5 biochar:water extract) 0.70 ± 0.01 dS m^−1^; CaCO_3_ 1.4 ± 0.03%; bulk density 0.20 ± 0.03 g cm^−3^; specific surface area 37.0 ± 2.13 m^2^ g^−1^; water holding capacity 350 ± 12.23%; moisture content 11.4 ± 1.09%; N 25.21 ± 2.91 mg kg^−1^; P 7.45 ± 0.83 mg kg^−1^; and K 13.21 ± 1.42 mg kg^−1^. During biochar preparation, it was grounded in a stainless steel mill and sieved through a ~2 mm mesh to eliminate outsized particles following air drying and then machinery raked for leveling. Through the plough practice, biochar was spread to each plot and mixed thoroughly with the surface layer of soil (0–20 cm depth) at a rate of 2.5 kg biochar m^−2^, which is equivalent to 10 t ha^−1^. 

### 4.4. Soil Physicochemical Properties

At wheat harvest, soil samples were collected at a 0–30 cm depth using an auger. Soil samples were air-dried and passed through a 2-mm sieve for chemical properties analysis. The EC (dS m^−1^) was measured in soil paste extract, while pH was determined in 1:2.5 soil: distilled water suspension. The EC_e_ was measured by EC-meter (Genway, UK), whereas pH was estimated by pH-meter (Genway, UK, relative error; ±0.05). The concentration (meq L^−1^) of Na^+^, K^+^, Ca^2+^, Mg^2+^ ions was measured in soil paste extract using an atomic absorption spectrophotometer (AAS, PERKIN ELMER 3300) with a detection limit of 100 ppb (Sparks et al., 1996). Exchangeable sodium percentage (ESP) was calculated according to the formula suggested by [56]:

ESP = 1.95 + 1.03 × SAR (R^2^ = 0.92),

where SAR (sodium adsorption ratio) was calculated using the following equation as described by [57]:

$$SAR=\left[Na^{+}\right]/\sqrt{\frac{\left(\left[Ca^{2}{}^{+}\right]+\;\left[Mg^{2}{}^{+}\right]\right)}{2}},$$

where Na^+^, Ca^2+^, and Mg^2+^ were expressed in meq L^−1^.

### 4.5. Physiological Measurements

Chlorophyll contents in the fresh leaves of wheat (0.5 g) at the anthesis stage were washed, cleaned, and extracted with 5 mL acetone (80%) at 0–4 °C and then centrifuged at 10,000× *g* for 5 min. Chlorophyll, a and b, was measured by the method recommended by [58]. A spectrophotometer (Hitachi-U2001, Tokyo, Japan) was used to record the absorbance of supernatant at 645 and 663 nm wavelengths.

Free proline content in the leaves was determined following the method of [59]. Leaf samples (0.5 g) at the anthesis stage were homogenized in 5 mL of sulfosalycylic acid (3%) using mortar and pestle. About 2 mL of extract was taken in the test tube, and to it, 2 mL of glacial acetic acid and 2 mL of ninhydrin reagent were added. The reaction mixture was boiled in a water bath at 100 °C for 30 min. After cooling the reaction mixture, 6 mL of toluene was added and then transferred to a separating funnel. After thorough mixing, the chromophore containing toluene was separated and absorbance read at 520 nm in a spectrophotometer against toluene blank. Proline concentration was determined using a calibration curve and expressed as μ mol proline g^−1^ FW.

Leaf relative water content: at the anthesis stage, five leaves detached from the stem were weighted to determine fresh weight (FW). Turgid weight (TW) was estimated after the leaves were kept floating in distilled water into a closed petri dish at 10 °C in the dark for 24 h and weighed again. Dry weight (DW) was determined for leaves samples after oven drying for 72 h at 80 °C. RWC was calculated using the following equation: LRWC (%) = [(FW − DW)/(TW − DW)] × 100 [60].

Stomatal conductance (g_s_) was measured on fully expanded three flag leaves at the anthesis stage from the abaxial surface as mmol H_2_O m^−2^ s^−1^ from three plants in each plot with a dynamic diffusion porometer (Delta-T AP4, Delta-T Devices Ltd, Cambridge, UK) at fine days. Two measurements from both adaxial and abaxial surfaces of the leaf were taken. It measured the fine days (following weather) every 4 or 7 days from booting untill harvest with a porometer [61]. Measurement in the top leave and front (r_a_) and back side (r_b_) of the center of the leaf. 

Total leaf conductance (r_l_) is 1/r_l_ = 1/r_a_ + 1/r_b_.

Electrolyte leakage (%) was measured in five fresh leaves at the anthesis stage. Twenty discs (1 cm^2^) were placed into flasks containing 25 mL deionized water; the flasks were shaken at ambient temperature (20 h) to facilitate electrolyte leakage from tissues. Initial electrical conductivity measurements were recorded. Flasks were then immersed in a hot water bath (80 °C) for 1 h to induce cell rupture. The samples were again placed on the shaker for 20 h at 21 °C. Final conductivity was measured for each flask. Electrolyte leakage (%) was calculated as follow: Initial conductivity/final conductivity × 100 [62].

The leaf photosynthetic rate (An) was measured at 10:00 to 13:00 a.m. on upper canopy of fully five mature leaves at anthesis stage using portable photosynthetic system (LiCor-6400, LI-Cor Bioscience, NE, USA). The measurements were performed on five leaves per plot at a CO_2_ concentration of 400 ppm with 1200 µmol m^−2^ s^−1^ photon flux density under 28.5 °C chamber temperature. The parameters An and g_s_ were used to calculate the intrinsic water use efficiency (WUE_i_; ratio An/g_s_). 

#### Leaf Na^+^ and K^+^ Determination

The dry five leaves at anthesis stage were ground and digested by HNO_3_. The concentration of Na^+^ and K^+^ was determined by an atomic absorption spectrometer (ICE3500, Thermo Fisher Scientific Inc., Waltham, MA, USA) [63].

### 4.6. Biochemical Analysis

In order to estimate the peroxidase (POX) and catalase (CAT) enzyme activities, five flag leaves of wheat were collected at 85 days after sowing and homogenized in a cooled 0.1 mol L^−1^ Tris-HCl buffer at pH 7.8 containing 1 mmol L^−1^ EDTA, 1 mmol L^−1^ dithiothreitol, and 5 mL of 4w/w polyvinyl pyrrolidone per one gram of fresh weight. Two mL reaction mixture consisting of 20 µL crude leaf extract, 660 µL potassium phosphate buffer (pH 7.0), 660 µL ascorbic acid solution, and 660 µL H_2_O_2_ was used to measure (POX) activity. Enzyme activity was tested by observing the ascorbate reduction through H_2_O_2_ at 290 nm for 3 min [64]. On the other hand, catalase (CAT) activity was extract by grinding 1 g of leaf tissues in 0.1 M sodium phosphate buffer at pH 7.1 in a porcelain mortar. Reaction mixture contained 25 mmol/L tris-acetate buffer (pH 7.0), 0.8 mM L^−1^ EDTA-Na, and 20 mM L^−1^ H_2_O_2_ at 25 °C. Enzyme activity was tested by observing H_2_O_2_ consumption at 240 nm for 3 min [65]. Enzymes activities were calculated in the form of µM H_2_O_2_ min^−1^ g^−1^ FW [66]. 

Peroxidase (POX) activity was measured according to the methodology of [67]. The mixture consisted of 2.9 mL of a 100 mM sodium phosphate buffer (pH 6.0) containing 0.25% (v/v) guaiacol and 100 mM H_2_O_2_. 100 µL of crude enzyme extract was added, the absorbance was recorded every 30 s at 470 nm for 3 min. The activity of the enzyme was recorded as min^−1^ g^−1^ fresh weight.

The superoxide dismutase (SOD) activity was measured by using the procedure given by [67] at 560 nm. Briefly, SOD was calculated through the prevention of nitro blue tetrazolium (NBT) at 560 nm as result of photochemical reduction.

Determination of lipid peroxidation was done according to [68], lipid peroxidation was measured as malondialdehyde (MDA) using spectrophotometer as follows: MDA (nmol g^−1^ FW) = [6.45 × (A532 − A600) − (0.56 × A450)] × V-1W, where V = volume (mL), W = weight (g).

#### Yield and Its Related Parameters

At maturity, ten samples from each experimental plot were randomly selected for counting 1000-grain weight, number of grains per spike and number of spikes per m^2^. In addition, at physiological maturity, 6 m^2^ area of each plot were manually harvested from the middle. The whole harvested plants of the 6 m^2^ were weighted to calculate the biological yield. Then, grains of the harvested plants were threshed with a thresher machine, dried in oven at + 85 °C for 24 h and then the weight of grain yield was measured. 

### 4.7. Nutrient Uptake

Uptake of N, P, and K were measured from multiplying a percentage of the specified element (nitrogen, phosphorus, potassium, and sodium) by grain and straw yield as a dry matter to calculate total nitrogen uptake (kg ha^−1^), total phosphorus uptake (kg ha^−1^), total potassium uptake (kg ha^−1^), and total sodium uptake (kg ha^−1^). Nitrogen element was determined by macro-Kjeldahl technique according to [69]. Phosphorus, potassium, and sodium elements were determined according to the flame photometer according to [63].

### 4.8. Statistical Analysis

Dependent variables were checked for normality and homoscedasticity and transformed as necessary. Data analysis was performed using Microsoft Excel 2003 (mean values ± standard deviation) and the SPSS 13.0 software package (SPSS Inc., Chicago, IL, USA). The analysis of variance using two-way ANOVA was conducted between treatments and growing season, while one-way ANOVA was applied to evaluate the differences among treatments within the same growing season. The experimental layout was a split-plot design with three repeats. Separation of means was performed by post hoc test (Tukey’s test), and significant differences were accepted at the levels *p* < 0.05, 0.01, and 0.001. 

## 5. Conclusions

Our findings exhibited that salt and water limitation induced a decrease in soil and physiological traits of wheat were increased by the exogenous application of SA alongside biochar treated soil. This increased impact of applied biochar on wheat production could be more efficacies through exogenous spraying of salicylic acid. While integrative use of biochar and SA were effective in declining electrolyte leakage, proline content, lipid peroxidation, and oxidative stress. To the best of our information, this is the initial investigation to testify the coupling application of biochar and SA on enhancing wheat yield under water deficit condition alongside saline sodic soil. This strategy might be a unique management for eco-friendly agriculture to augment soil health and wheat productivity. 

## Figures and Tables

**Table 1:** Soil chemical properties at the harvest of wheat plants irrigated with (50%, 70%, and 90% depletion of available soil moisture (DAM)) in saline sodic soil treated by salicylic acid and biochar during two growing seasons.

Year	Treatments (Ts)		pH ^¥^	EC ^§^	ESP ^#^	Na^+¤^	K^+^	Ca^2+^	Mg^2+^
	Water Ts	Soil and Foliar Ts		(dS m^−1^)	(%)	(meq L^−1^)	(meq L^−1^)	(meq L^−1^)	(meq L^−1^)
2018/2019	50% DAM	Control	8.03 ±0.00gh	3.57 ±0.03ef	11.03 ±0.22ef	16.77 ±0.85ef	0.42 ±0.02cd	14.88 ±0.05d	6.90 ±0.04d
		SA ^†^	8.04 ±0.01h	3.55 ±0.06fg	10.97 ±0.12fg	15.92 ±1.05fg	0.43 ±0.05c	15.10 ±0.09cd	7.09 ±0.03cd
		BC ^‡^	8.00 ±0.01i	3.20 ±0.04g	8.01 ±0.33g	12.84 ±0.02g	0.46 ±0.04b	17.49 ±0.18b	7.28 ±0.02b
		SA+BC	7.98 ±0.01j	3.04 ±0.08h	7.54 ±0.11h	10.24 ±0.11h	0.48 ±0.03a	19.32 ±0.22a	7.41 ±0.01a
	70% DAM	Control	8.10 ±0.03cd	3.95 ±0.07c	15.23 ±0.36c	20.55 ±1.08c	0.37 ±0.00ef	10.57 ±0.15fg	6.69 ±0.09fg
		SA	8.08 ±0.03e	3.88 ±0.04d	15.05 ±0.35d	19.78 ±1.07d	0.38 ±0.01e	10.95 ±0.18f	6.72 ±0.06f
		BC	8.05 ±0.02f	3.64 ±0.05e	12.87 ±0.24e	17.23 ±0.99e	0.41 ±0.82d	13.45 ±0.08de	6.97 ±0.05de
		SA+BC	8.02 ±0.01g	3.52 ±0.02f	10.91 ±0.15f	16.07 ±0.87f	0.43 ±0.03c	15.85 ±0.07c	7.02 ±0.08c
	90% DAM	Control	8.17 ±0.02a	4.74 ±0.06a	22.93 ±0.23a	27.44 ±0.98a	0.33 ±0.02hi	6.80 ±0.12hi	6.08 ±0.02hi
		SA ^†^	8.16 ±0.02b	4.68 ±0.05ab	21.49 ±0.35ab	26.64 ±0.88ab	0.34 ±0.01h	7.08 ±0.18h	6.19 ±0.03h
		BC ^‡^	8.11 ±0.01c	4.15 ±0.03b	17.92 ±0.24b	21.84 ±0.75b	0.36 ±0.03g	9.16 ±0.23g	6.54 ±0.08g
		SA+BC	8.09 ± 0.02d	3.89 ±0.08cd	15.19 ±0.15cd	19.94 ±1.05cd	0.38 ±0.02f	11.24 ±0.25e	6.72 ±0.07e
2019/2020	50% DAM	Control	8.01 ±0.00gh	3.54 ±0.03ef	10.99 ±0.22ef	16.72 ±0.85ef	0.44 ±0.02cd	14.94 ±0.05d	6.96 ±0.04d
		SA ^†^	8.02 ±0.01h	3.52 ±0.06fg	10.93 ±0.12fg	15.87 ±1.05fg	0.45 ±0.05c	15.16 ±0.09cd	7.15 ±0.03cd
		BC ^‡^	7.98 ±0.01i	3.17 ±0.04g	7.97 ±0.33g	12.79 ±0.02g	0.48 ±0.04b	17.55 ±0.18b	7.34 ±0.02b
		SA+BC	7.87 ±0.01j	3.01 ±0.08h	7.50 ±0.11h	10.19 ±0.11h	0.50 ±0.03a	19.38 ±0.22a	7.47 ±0.01a
	70% DAM	Control	8.08 ±0.03cd	3.92 ±0.07c	15.19 ±0.36c	20.50 ±1.08c	0.39 ±0.00ef	10.63 ±0.15fg	6.75 ±0.09fg
		SA	8.06 ±0.03e	3.85 ±0.04d	15.01 ±0.35d	19.73 ±1.07d	0.40 ±0.01e	11.01 ±0.18f	6.78 ±0.06f
		BC	8.03 ±0.02f	3.61 ±0.05e	12.83 ±0.24e	17.17 ±0.99e	0.43 ±0.82d	13.51 ±0.08de	7.03 ±0.05de
		SA+BC	8.00 ±0.01g	3.49 ±0.02f	10.87 ±0.15f	16.02 ±0.87f	0.45 ±0.03c	15.91 ±0.07c	7.08 ±0.08c
	90% DAM	Control	8.15 ±0.02a	4.71 ±0.06a	22.89 ±0.23a	27.39 ±0.98a	0.35 ±0.02hi	6.86 ±0.12hi	6.14 ±0.02hi
		SA ^†^	8.14 ±0.02b	4.65 ±0.05ab	21.45 ±0.35ab	26.59 ±0.88ab	0.36 ±0.01h	7.14 ±0.18h	6.25 ±0.03h
		BC ^‡^	8.09 ±0.01c	4.12 ±0.03b	17.88 ±0.24b	21.79 ±0.75b	0.38 ±0.03g	9.22 ±0.23g	6.60 ±0.08g
		SA+BC	8.07 ±0.02d	3.86 ±0.08cd	15.15 ±0.15cd	19.89 ±1.05cd	0.40 ±0.02f	11.30 ±0.25e	6.78 ±0.07e
F-test									
W			***	***	***	***	***	***	***
SF			***	***	***	***	***	***	***
W × SF			***	***	***	ns	ns	ns	***

^¥^ pH is measured in soil:distilled water suspension at 1:2.5 ratio. Water treatments (W); soil and foliar treatments (SF). ^§^ EC (electrical conductivity) is measured in soil:distilled water extract of 1:5. ^#^ ESP (exchangeable sodium percentage). Depletion of available moisture (DAM). Ions (Na^+^, K^+^, Ca^2+^, and Mg^2+^) are measured in soil:distilled water extract of 1:5. ^†^ Salicylic acid (SA) is added at the rate of 192 g ha^−1^; ^‡^ Biochar (BC) is added at the rate of 10 t ha^−1^. Means of the same growing season designated with different letters indicate significant differences among treatments according to the Tukey’s test (*P* < 0.05). Values are means ± standard deviation (SD) from three replicates (Means ± SD). *** and ns denote significance at *P* < 0.001 and non-significant.

**Table 2:** The percent of Na^+^ and K^+^ in the leaves of wheat plants irrigated with (50%, 70%, and 90% DAM) in saline sodic soil treated by salicylic acid and biochar during two growing seasons.

		2018/2019		2019/2020	
Water Treatments	Soil and Foliar Treatments	Na^+^ (%)	K^+^ (%)	Na^+^ (%)	K^+^ (%)
50% DAM	Control	1.79 ± 0.02e	1.19 ± 0.01cd	1.81 ± 0.01ef	1.15 ± 0.03cd
	SA ^†^	1.63 ± 0.02f	1.32 ± 0.02bc	1.75 ± 0.02g	1.27 ± 0.02bc
	BC ^‡^	1.59 ± 0.01g	1.38 ± 0.02b	1.69 ± 0.01gh	1.32 ± 0.01b
	SA + BC	1.38 ± 0.02h	1.49 ± 0.02a	1.52 ± 0.02h	1.39 ± 0.01a
70% DAM	Control	2.25 ± 0.01c	0.95 ± 0.01g	2.21 ± 0.02c	0.89 ± 0.00ef
	SA	2.09 ± 0.02d	1.08 ± 0.02ef	2.02 ± 0.02d	0.98 ± 0.01de
	BC	2.02 ± 0.02de	1.17 ± 0.02e	1.88 ± 0.03e	1.08 ± 0.02d
	SA + BC	1.76 ± 0.03ef	1.22 ± 0.01c	1.79 ± 0.03f	1.17 ± 0.02c
	Control	2.86 ± 0.03a	0.71 ± 0.01j	2.74 ± 0.01a	0.65 ± 0.01h
90% DAM	SA	2.67 ± 0.01b	0.82 ± 0.02i	2.35 ± 0.02b	0.73 ± 0.02g
	BC	2.52 ± 0.02bc	0.88 ± 0.02h	2.27 ± 0.02bc	0.81 ± 0.02f
	SA + BC	2.13 ± 0.01cd	0.99 ± 0.02f	2.19 ± 0.03cd	0.92 ± 0.01e
**F-test**					
W		***	***	***	***
SF		***	***	***	***
W × SF		ns	***	***	***

Water treatments (W); soil and foliar treatments (SF). Depletion of available moisture (DAM). ^†^ Salicylic acid (SA) is added at the rate of 192 g ha^−1^; ^‡^ Biochar (BC) is added at the rate of 10 t ha^−^^1^; Means of the same growing season designated with different letters indicate significant differences among treatments according to the Tukey’s test (*P* < 0.05). Values are means ± standard deviation (SD) from three replicates (Means ± SD). *** and ns denote significance at *P* < 0.001 and non-significant.

**Table 3:** Physiological properties of wheat plants irrigated with (50%, 70%, and 90% DAM) in saline sodic soil treated by salicylic acid and biochar during two growing seasons.

Year	Water Treatments	Soil and Foliar Treatments	Chlorophyll a (mg g^−1^ FW)	Chlorophyll b (mg g^−1^ FW)	Proline (µ mol g^−1^ FW)	RWC ^#^ (%)	EL ^¥^ (%)
2018/2019	50% DAM	Control	1.32 ± 0.02c	0.55 ± 0.03cd	7.44 ± 0.03e	88.55 ± 1.54de	19.25 ± 1.18h
		SA ^†^	1.52 ± 0.05b	0.67 ± 0.04b	7.28 ± 0.02fg	93.25 ± 1.74b	15.48 ± 1.02k
		BC ^‡^	1.48 ± 0.04bc	0.64 ± 0.04bc	7.36 ± 0.03f	91.05 ± 1.65c	16.65 ± 1.05j
		SA + BC	1.68 ± 0.03a	0.76 ± 0.04a	7.17 ± 0.01h	94.98 ± 1.85a	13.47 ± 1.22l
	70% DAM	Control	1.13 ± 0.01g	0.37 ± 0.00ef	8.54 ± 0.02c	82.95 ± 1.48gh	27.14 ± 1.12e
		SA	1.26 ± 0.03e	0.51 ± 0.02d	7.55 ± 0.01de	87.47 ± 1.95e	20.65 ± 1.13g
		BC	1.23 ± 0.02f	0.48 ± 0.01de	8.12 ± 0.01d	85.58 ± 1.65f	21.89 ± 1.15f
		SA + BC	1.37 ± 0.02d	0.58 ± 0.03c	7.35 ± 0.00ef	89.09 ± 1.14d	17.48 ± 1.16i
	90% DAM	Control	1.01 ± 0.02k	0.24 ± 0.00h	12.47 ± 0.01a	75.42 ± 1.58j	45.63 ± 1.02a
		SA	1.09 ± 0.03i	0.34 ± 0.01f	9.32 ± 0.03bc	80.85 ± 1.36h	32.74 ± 1.05c
		BC	1.05 ± 0.01j	0.31 ± 0.01g	9.45 ± 0.02b	78.06 ± 1.47i	36.55 ± 1.09b
		SA + BC	1.18 ± 0.01h	0.39 ± 0.02e	8.45 ± 0.02cd	84.25 ± 1.65g	25.83 ± 1.08d
2019/2020	50% DAM	Control	1.43 ± 0.01d	0.69 ± 0.04c	7.23 ± 0.04e	85.01 ± 1.95de	20.69 ± 1.05h
		SA ^†^	1.55 ± 0.05b	0.81 ± 0.03ab	6.65 ± 0.02g	88.36 ± 1.45b	16.58 ± 1.07k
		BC ^‡^	1.51 ± 0.03bc	0.78 ± 0.04b	6.87 ± 0.03f	85.97 ± 1.65c	17.74 ± 1.13j
		SA + BC	1.63 ± 0.05a	0.93 ± 0.02a	6.14 ± 0.01h	91.66 ± 1.53a	14.98 ± 1.11l
	70% DAM	Control	1.23 ± 0.03gh	0.46 ± 0.03f	8.42 ± 0.02c	80.22 ± 1.12g	28.74 ± 1.12e
		SA	1.36 ± 0.02e	0.64 ± 0.04d	7.78 ± 0.01de	84.12 ± 1.14e	21.45 ± 1.15g
		BC	1.31 ± 0.03f	0.59 ± 0.03e	8.07 ± 0.02d	82.05 ± 1.85f	22.63 ± 1.14f
		SA + BC	1.48 ± 0.02c	0.71 ± 0.04cd	7.02 ± 0.02ef	86.47 ± 1.75d	18.21 ± 1.10i
	90% DAM	Control	1.05 ± 0.01j	0.28 ± 0.01i	12.42 ± 0.03a	76.25 ± 1.74j	46.21 ± 1.09a
		SA	1.22 ± 0.02h	0.40 ± 0.02h	9.36 ± 0.04bc	79.23 ± 1.85h	33.75 ± 1.08c
		BC	1.14 ± 0.02i	0.37 ± 0.01hi	9.44 ± 0.03b	77.05 ± 1.96i	37.22 ± 1.05b
		SA + BC	1.28 ± 0.03g	0.49 ± 0.01g	8.22 ± 0.04cd	82.66 ± 1.32fg	26.45 ± 1.11d
**F-test**							
W			***	***	***	***	**
SF			***	***	***	***	***
W × SF			***	***	***	***	ns

^#^ Relative water content; ^¥^ electrolyte leakage. Depletion of available moisture (DAM). Water treatments (W); soil and foliar treatments (SF). ^†^ Salicylic acid (SA) is added at the rate of 192 g ha^−1^; ^‡^ Biochar (BC) is added at the rate of 10 t ha^−1^. Means of the same growing season designated with different letters indicate significant differences among treatments according to the Tukey’s test (*P* < 0.05). Values are means ± standard deviation (SD) from three replicates (Means ± SD). ***, **, and ns denote significance at *P* < 0.001, *P* < 0.01, and non-significant, respectively.

**Table 4:** Yield, yield components, and nutrient uptake of wheat plants irrigated with (50%, 70%, and 90% DAM) in saline sodic soil treated by salicylic acid and biochar during two growing seasons.

Year	Treatments (Ts)		Grains Per Spike	1000-Grain Weight	Grain Yield	N Uptake	P Uptake	K Uptake
	Water Ts	Soil and Foliar Ts	(n)	(g)	(ton/ha)	(kg ha^−1^)	(kg ha^−1^)	(kg ha^−1^)
2018/2019	50% DAM	Control	50.75 ± 1.21d	52.50 ± 0.75d	4.74 ± 0.04cd	86.5 ± 1.53e	47.6 ± 1.15e	130.5 ± 2.74e
		SA ^†^	53.66 ± 1.20c	53.98 ± 0.84bc	5.02 ± 0.03bc	93.8 ± 1.57c	54.1 ± 1.14c	140.7 ± 2.95c
		BC ^‡^	55.69 ± 1.22b	54.99 ± 0.25b	5.38 ± 0.05b	95.9 ± 1.66b	59.5 ± 1.22b	147.8 ± 2.68b
		SA+BC	57.96 ± 1.25a	56.44 ± 0.36a	6.67 ± 0.08a	103.8 ± 1.62a	62.8 ± 1.25a	163.7 ± 2.11a
	70% DAM	Control	45.24 ± 1.24h	48.65 ± 0.22g	3.95 ± 0.03ef	69.4 ± 1.44i	35.0 ± 1.23gh	102.4 ± 3.65h
		SA	47.66 ± 1.1.18f	50.32 ± 0.54ef	4.34 ± 0.07d	76.4 ± 1.45g	38.4 ± 1.18f	117.6 ± 2.36fg
		BC	49.47 ± 1.19ef	51.66 ± 0.48e	4.54 ± 0.08d	80.3 ± 1.48f	39.2 ± 1.18f	120.9 ± 3.22f
		SA+BC	51.23 ± 1.15c	53.21 ± 0.65c	4.88 ± 0.06c	88.9 ± 1.52d	50.0 ± 1.19d	133.5 ± 3.45d
	90% DAM	Control	39.65 ± 1.25h	45.24 ± 0.50i	2.99 ± 0.06h	45.7 ± 1.56k	21.6 ± 1.12j	79.3 ± 2.35k
		SA	42.96 ± 1.26gh	46.89 ± 0.65h	3.42 ± 0.05g	61.0 ± 1.58j	28.3 ± 1.22ij	90.5 ± 2.36j
		BC	44.35 ± 1.22g	47.03 ± 0.62h	3.78 ± 0.02f	62.3 ± 1.59j	30.4 ± 1.25i	94.5 ± 2.45i
		SA+BC	46.78 ± 1.25e	48.99 ± 0.84f	4.05 ± 0.01e	72.4 ± 1.58h	36.5 ± 1.24g	104.4 ± 2.44g
2019/2020	50% DAM	Control	49.65 ± 1.17d	53.75 ± 0.74d	5.18 ± 0.06cd	81.3 ± 1.69e	50.4 ± 1.21d	136.7 ± 2.45de
		SA ^†^	52.75 ± 1.14c	55.74 ± 0.48bc	5.54 ± 0.04bc	89.4 ± 1.45c	55.7 ± 1.19c	150.9 ± 2.65c
		BC ^‡^	54.25 ± 1.12b	56.48 ± 0.65b	5.87 ± 0.03b	95.4 ± 1.55b	57.7 ± 1.18b	159.4 ± 2.55b
		SA+BC	56.74 ± 1.06a	57.98 ± 0.24a	6.23 ± 0.02a	111.0 ± 1.89a	62.4 ± 1.18a	174.4 ± 2.85a
	70% DAM	Control	45.88 ± 1.09g	49.07 ± 0.25ef	3.92 ± 0.05f	64.8 ± 1.47i	38.0 ± 1.17g	105.5 ± 2.36g
		SA	47.99 ± 1.05e	51.74 ± 0.14de	4.36 ± 0.04d	70.4 ± 1.56g	44.5 ± 1.23ef	119.7 ± 2.24ef
		BC	48.87 ± 1.07e	52.42 ± 0.23d	4.87 ± 0.01d	77.9 ± 1.44f	45.9 ± 1.25e	122.4 ± 2.65e
		SA+BC	50.75 ± 1.18c	54.44 ± 0.65c	5.33 ± 0.05c	83.4 ± 1.58d	52.4 ± 1.24cd	139.7 ± 2.15d
	90% DAM	Control	39.89 ± 1.11i	44.55 ± 0.86g	3.02 ± 0.07i	47.2 ± 1.63k	22.4 ± 1.24j	80.4 ± 2.65i
		SA	41.25 ± 1.15g	46.25 ± 0.47fg	3.43 ± 0.06gh	58.3 ± 1.60jk	30.7 ± 1.20i	93.5 ± 2.45hi
		BC	43.22 ± 1.18gh	47.02 ± 0.63f	3.78 ± 0.03g	60.8 ± 1.55j	34.5 ± 1.14h	96.1 ± 2.75h
		SA+BC	46.22 ± 1.19f	49.78 ± 0.54e	4.09 ± 0.02e	66.7 ± 1.54h	40.8 ± 1.18f	108.5 ± 2.45f
F-test								
W			***	***	***	***	***	***
SF			***	**	***	***	ns	**
W × SF			***	**	***	**	*	**

Water treatments (W); soil and foliar treatments (SF). Depletion of available moisture (DAM). ^†^ Salicylic acid (SA) is added at the rate of 192 g ha^−1^; ^‡^ Biochar (BC) is added at the rate of 10 t ha^−1^; Means of the same growing season designated with different letters indicate significant differences among treatments according to the Tukey’s test (*P* < 0.05). Values are means ± standard deviation (SD) from three replicates (Means ± SD). ***, **, *, and ns denote significance at *P* < 0.001, *P* < 0.01, *P* < 0.05, and non-significant, respectively.

**Table 5:** Meteorological data for Sakha Station during 2018/2019 and 2019/2020 growing seasons.

**Year** **Month**	**2018/2019**				**2019/2020**			
	**Temperature (°C)**		**Preceptation (mm)**	**RH ^ώ^ (%)**	**Temperature (°C)**		**Preceptation (mm)**	**RH ^ώ^ (%)**
	max	min			max	min		
Dec	25.9	12.7	1.08	33.3	22.7	11.2	0.62	30.1
Jan	24.5	11.4	2.07	45.4	19.8	10.0	2.24	41.7
Feb	22.7	10.1	5.35	43.5	21.2	9.3	5.78	40.5
Mar	24.3	12.9	0.65	42.9	23.2	11.2	0.51	43.7
April	25.2	13.7	0.00	50.8	26.1	15.5	0.00	50.6
May	28.8	17.6	0.00	60.7	29.1	16.7	0.00	62.5

*^ώ^* relative humidity.

**Table 6:** Physicochemical characteristics of the experimental soil before planting in the two growing seasons 2018/2019 and 2019/2020.

Character	2018/2019	2019/2020
pH (1:2.5 soil:water suspension)	8.17 ± 0.03 ^†^	8.11 ± 0.01
Electrical conductivity (EC, dS m^−1^) ^¥^	4.24 ± 0.02	4.09 ± 0.01
Soil organic matter (g kg^−1^)	10.9 ± 0.02	12.2 ± 0.02
ESP ^#^ (%)	19.88 ± 0.39	17.37 ± 0.11
Particle size distribution (%)		
Sand	28.34 ± 1.75	25.32 ± 1.75
Silt	23.45 ± 2.03	26.44 ± 1.55
Clay	48.21 ± 2.14	48.24 ± 2.12
Texture grade	clayey	clayey
Soluble cations (meq L^−1^) ^¥^		
Ca2+	7.93 ± 0.84	8.75 ± 0.74
Mg2+	4.02 ± 1.08	3.95 ± 1.21
Na^+^	24.02 ± 2.02	20.56 ± 3.02
K^+^	0.52 ± 0.01	0.48 ± 0.11
SAR ^®^ (%)	9.22 ± 0.02	8.35 ± 0.13
Soluble anions (meq L^−1^) ^¥^		
CO_3_^− −^	nd ^‡^	nd
HCO_3_^−^	4.01 ± 0.44	4.03 ± 0.58
Cl^−^	25.89 ± 1.33	21.98 ± 1.24
SO_4_^− −^	16.29 ± 3.10	12.97 ± 3.09
Available macronutrients (mg kg^−1^)		
N	9.98 ± 0.54	11.44 ± 1.44
P	8.97 ± 1.26	9.74 ± 1.32
K	367 ± 25.38	392 ± 24.45

^†^ Standard deviation; ^‡^ not detected; ^¥^ measured in soil paste extract; ^#^ exchangeable sodium percentage, ^®^sodium adsorption ratio.

**Table 7:** Soil moisture constants before planting in the two growing seasons 2018/2019 and 2019/2020.

Year	Soil Depth (cm)	FC ^©^ (%)	PWP ^£^ (%)	ASW ^∞^ (%)	BD ^±^ (g cm^−3^)
2018/2019	0–20	41.29 ± 0.01	21.24 ± 0.03	20.05 ± 0.03	1.42 ± 0.02
	20–40	38.48 ± 0.02	21.55 ± 0.02	16.93 ± 0.02	1.44 ± 0.04
	40–60	37.67 ± 0.03	20.45 ± 0.04	17.22 ± 0.04	1.47 ± 0.03
2019/2020	0–20	42.16 ± 0.06	18.63 ± 0.03	23.53 ± 0.01	1.38 ± 0.05
	20–40	41.35 ± 0.05	19.75 ± 0.03	21.60 ± 0.02	1.39 ± 0.03
	40–60	41.54 ± 0.04	19.89 ± 0.02	21.65 ± 0.05	1.43 ± 0.02

^©^ field capacity, ^£^ permanent wilting point,^**∞**^ available soil water, ^±^ bulk density.

## References

[1] Wang X., Yang J., Liu G., Yao R., Yu S. (2015). Impact of irrigation volume and water salinity on winter wheat productivity and soil salinity distribution. Agric. Water Manag..

[2] Attia A., Rajan N., Xue Q., Nair S., Ibrahim A., Hays D. (2016). Application of DSSAT-CERES-Wheat model to simulate winter wheat response to irrigation management in the Texas High Plains. Agric. Water Manag..

[3] FAOSTAT Food and agriculture organization of the United Nations statistics division. http://faostat.fao.org/site/567/DesktopDefault.aspx.

[4] Hafez E.M., Kobata T. (2012). The effect of different nitrogen sources from urea and ammonium sulfate on the spikelet number in Egyptian spring wheat cultivars on well watered pot soils. Plant Prod. Sci..

[5] MALR (Ministry of Agriculture and Land Reclamation) (2009). Sustainable Agricultural Development Strategy towards 2030.

[6] FAO (2019). The Future of Food and Agriculture: Trends and Challenges.

[7] Tari A.F. (2016). The effects of different deficit irrigation strategies on yield, quality, and water-use efficiencies of wheat under semi-arid conditions. Agric. Water Manag..

[8] Boguszewska D., Zagdańska B. (2012). ROS as signaling molecules and enzymes of plant response to unfavorable environmental conditions. Oxidative Stress–Molecular Mechanisms and Biological Effects.

[9] Hafez E.M., Omara A., El-Esawi M. (2020). Minimizing hazard impacts of soil salinity and water stress on wheat plants by integrated soil application of vermicompost and biochar. Physiologia Plantarum.

[10] Hafez E., Omara A.E.D., Ahmed A. (2019). The Coupling Effects of Plant Growth Promoting Rhizobacteria and Salicylic Acid on Physiological Modifications, Yield Traits, and Productivity of Wheat under Water Deficient Conditions. Agronomy.

[11] Hafez E.M., Gharib H.S. (2016). Effect of exogenous application of ascorbic acid on physiological and biochemical characteristics of wheat under water stress. Int. J. Plant Prod..

[12] Kamara M.M., Rehan M., Ibrahim K.M., Alsohim A.S., Elsharkawy M.M., Kheir A.M.S., Hafez E.M., El-Esawi M.A. (2020). Genetic Diversity and Combining Ability of White Maize Inbred Lines under Different Plant Densities. Plants.

[13] Zhu J.K. (2016). Abiotic Stress Signaling and Responses in Plants. Cell.

[14] Ding Z., Kheir A.M.S., Ali O., Hafez E.M., Elshamey E.A., Zhou Z., Wang B., Lin X., Ge Y., Fahmy A.E. (2020). Vermicompost and deep tillage system as an environmental method to improve saline-alkaline soils and wheat productivity. J. Environ. Manag..

[15] Chai Q., Gan Y.T., Zhao C., Xu H.L., Waskom R.M., Niu Y.N., Siddique K.H.M. (2016). Regulated deficit irrigation for crop production under drought stress. Rev. Agron. Sustain. Dev..

[16] Wang W., Vinocur B., Altman A. (2003). Plant responses to drought, salinity and extreme temperatures: Towards genetic engineering for stress tolerance. Planta.

[17] Asadi M., Heidari M.A., Kazemi M., Filinejad A.R. (2013). Salicylic acid induced changes in some physiological parameters in chickpea (Cicer arietinum L.) under salt stress. J. Agric. Sci. Technol..

[18] Hasanuzzaman M., Matin M., Fardus J., Hasanuzzaman M., Hossain M., Parvin K. (2019). Foliar application of salicylic acid improves growth and yield attributes by upregulating the antioxidant defense system in Brassica campestris plants grown in lead-amended soils. Acta Agrobot..

[19] Gunes A., Inal A., Alpaslan M., Cicek N., Guneri E., Eraslan F. (2007). Effects of exogenously applied salicylic acid on the induction of multiple stress tolerance and mineral nutrition in maize (*Zea mays* L.). Arch. Agron. Soil Sci..

[20] Kang G., Li G., Liu G., Xu W., Peng X., Wang C., Zhu Y., Guo T. (2013). Exogenous salicylic acid enhances wheat drought tolerance by influence on the expression of genes related to ascorbate- glutathione cycle. Biol. Plants.

[21] Mutlu S., Ökke¸s A., Nalbanto˘glu B., Mete E. (2016). Exogenous salicylic acid alleviates cold damage by regulating antioxidative system in two barley (Hordeum vulgare L.) cultivars. Front. Life Sci..

[22] Hafez E., Farig M. (2019). Efficacy of salicylic acid as a cofactor for ameliorating effects of water stress and enhancing wheat yield and water use efficiency in saline soil. Int. J. Plant Prod..

[23] Rahmani I., Ahmadi N., Ghanati F., Sadeghi M. (2015). Effects of salicylic acid applied pre-or post-transport on post-harvest characteristics and antioxidant enzyme activity of gladiolus cut flower spikes. N. Z. J. Crop Hortic. Sci..

[24] Razmi N., Ebadi A., Daneshian J., Jahanbakhsh S. (2017). Salicylic acid induced changes on antioxidant capacity, pigments and grain yield of soybean genotypes in water deficit condition. J. Plant Int..

[25] Akhtar S.S., Andersen M.N., Liu F. Residual effects of biochar on improving growth, physiology and yield of wheat under salt stress. Agric. Water Manag..

[26] Akhtar S.S., Andersen M.N., Liu F. (2015). Biochar Mitigates Salinity Stress in Potato. J. Agron. Crop Sci..

[27] Thi N., Xu C.-Y., Tahmasbian I., Che R., Xu Z., Zhou X., Wallace H.M., Bai S.H. (2017). Effects of biochar on soil available inorganic nitrogen: A review and meta-analysis. Geoderma.

[28] Leng L., Huang H., Li H., Li J., Zhou W. (2019). Biochar stability assessment methods: A review. Sci. Total Environ..

[29] Seleiman M.F., Refay Y., Al-Suhaibani N., Al-Ashkar I., El-Hendawy S., Hafez E.M. (2019). Integrative Effects of Rice-Straw Biochar and Silicon on Oil and Seed Quality, Yield and Physiological Traits of Helianthus annuus L. Grown under Water Deficit Stress. Agronomy.

[30] Yang A., Akhtar S.S., Li L., Fu Q., Li Q., Naeem M.A., He X., Zhang Z., Jacobsen S.-E. (2020). Biochar Mitigates Combined Effects of Drought and Salinity Stress in Quinoa. Agronomy.

[31] Zheng W., Sharma B.K., Rajagopalan N. (2010). Using Biochar as a Soil Amendment for Sustainable Agriculture.

[32] Hafez E.M., Alsohim A.S., Farig M., Omara A.E.D., Rashwan E., Kamara M.M. (2019). Synergistic Effect of Biochar and Plant Growth Promoting Rhizobacteria on Alleviation of Water Deficit in Rice Plants under Salt-Affected Soil. Agronomy.

[33] Dahlawi S., Naeem A., Rengel Z., Naidu R. (2018). Biochar application for the remediation of salt-affected soils: Challenges and opportunities. Sci. Total Environ..

[34] Hussain M., Farooq M., Nawaz A., Al-Sadi A.M., Solaiman Z.M., Alghamdi S.S., Ammara U., Ok Y.S., Siddique K.H.M. (2017). Biochar for crop production: Potential benefits and risks. J. Soils Sediments.

[35] Duarte D.J., Glaser B., Cerri P., Eduardo C. (2019). Effect of Biochar Particle Size on Physical, Hydrological and Chemical Properties of Loamy and Sandy Tropical Soils. Agronomy.

[36] Haider G., Steffens D., Moser G., Müller C., Kammann C.I. (2017). Biochar reduced nitrate leaching and improved soil moisture content without yield improvements in a four-year field study. Agric. Ecosyst. Environ..

[37] Hayat Q., Hayat S., Irfan M., Ahmad A. (2010). Effect of exogenous salicylic acid under changing environment: A review. Environ. Exp. Bot..

[38] Dzvene A.R., Chiduza C., Mnkeni P.N.S., Peter P.C. (2019). Characterization of livestock biochars and their effect on selected soil properties and maize early growth stage in soils of Eastern Cape province, South Africa. S. Afr. J. Plant Soil.

[39] Omondi M.O., Xia X., Nahayo A., Liu X., Korai P.K., Pan G. (2016). Quantification of biochar effects on soil hydrological properties using meta-analysis of literature data. Geoderma.

[40] Ijaz M., Tahir M., Shahid M., Ul-Allah S., Sattar A., Sher A., Mahmood K.M. (2019). Combined application of biochar and PGPR consortia for sustainable production of wheat under semiarid conditions with a reduced dose of synthetic fertilizer. Braz. J. Microbiol..

[41] Yu O.Y., Harper M., Hoepfl M., Domermuth D. (2017). Characterization of biochar and its effects on the water holding capacity of loamy sand soil: Comparison of hemlock biochar and switchblade grass biochar characteristics. Environ. Prog. Sustain. Energy.

[42] Jini D., Joseph B. (2017). Physiological mechanism of salicylic acid for alleviation of salt stress in rice. Rice Sci..

[43] Jones H.G. (1998). Stomatal control of photosynthesis and transpiration. J. Exp. Bot..

[44] Li T., Hu Y., Du X., Tang H., Shen C. (2014). Salicylic acid alleviates the adverse effects of salt stress in Torreya grandis cv. Merrillii seedlings by activating photosynthesis and enhancing antioxidant systems. PLoS ONE.

[45] Hussain M., Malik M.A., Farooq M., Ashraf M.Y., Cheema A. (2008). Improving Drought tolerance by exogenous application of glycinebetaine and salicylic acid in sunflower. J. Agron. Crop Sci..

[46] Hafez E.M., Seleiman M.F. (2017). Response of barley quality traits, yield and antioxidant enzymes to water-stress and chemical inducers. Int. J. Plant Prod..

[47] Arfan M., Athar H.R., Ashraf M. (2007). Does exogenous application of salicylic acid through the rooting medium modulate growth and photosynthetic capacity in two differently adapted spring wheat cultivars under salt stress?. J. Plant Physiol..

[48] Hafez E.M., Ragab A.Y., Kobata T. (2014). Water-Use Efficiency and Ammonium-N Source Applied of Wheat under Irrigated and Desiccated Conditions. Int. J. Plant Soil Sci..

[49] Hayat S., Hayat Q., Alyemeni M.N., Wani A.S., Pichtel J., Ahmad A. (2012). Role of proline under changing environments: A review. Plant Signal. Behav..

[50] Pirasteh-Anosheh H., Ranjbar G., Emam Y., Ashraf M. (2014). Salicylic-acid-induced recovery ability in salt-stressed Hordeum vulgare plants. Turk. J. Bot..

[51] Gharib H., Hafez E., El Sabagh A. (2016). Optimized Potential of Utilization Efficiency and Productivity in Wheat by Integrated Chemical Nitrogen Fertilization and Stimulative Compounds. Cercetari Agronomice Moldova.

[52] Bistgani Z.E., Siadat S.A., Bakhshandeh A., Pirbalouti A.G., Hashemi M. (2017). Morpho-physiological and phytochemical traits of (*Thymus daenensis* Celak.) in response to deficit irrigation and chitosan application. Acta Physiol. Plant.

[53] Hafez E.M. (2016). Influence of salicylic acid on ion distribution, enzymatic activity and some agromorphological characteristics of wheat under salt-affected soil. Egyptian J. Agron..

[54] Xie W.J., Wu L.F., Zhang Y.P., Wu T., Li X.P., Ouyang Z. (2017). Effects of straw application on coastal saline topsoil salinity and wheat yield trend. Soil Till. Res..

[55] Kheir A.S., Abou elsoud H.M., Hafez E.M., Ali O.A. (2019). Integrated effect of nano-Zn, nano-Si, and drainage using crop straw-filled ditches on saline sodic soil properties and rice productivity. Arab. J. Geosci..

[56] Seilsepour M., Rashidi M. (2008). Prediction of soil cation exchange capacity based on some soil physical and chemical properties. World Appl. Sci. J..

[57] Richards L.A., U.S.D.A (1954). Diagnosis and Improvement of Saline and Alkali Soils. Agriculture Handbook No. 60.

[58] Arnon D.I. (1949). Copper enzymes in isolated chloroplasts. Polyphenoloxidase in Beta vulgaris. Plant Physiol..

[59] Bates L.S., Waldren R.P., Teare I.D. (1973). Rapid determination of free proline for water-stress studies. Plant Soil.

[60] Sanchez F.J., de Andrés E.F., Tenorio J.L., Ayerbe L. (2004). Growth of epicotyls, turgor maintenance and osmotic adjustment in pea plants (Pisum sativum L.) subjected to water stress. Field Crops Res..

[61] Izanloo A., Anthony G., Thorsten S. (2008). Different mechanisms of adaptation to cyclic water stress in two South Australian bread wheat cultivars. J. Exp. Bot..

[62] Nishiyama R., Watanabe Y., Fujita Y., Le D.T., Kojima M., Werner T., Vankova R., Yamaguchi-Shinozaki K., Shinozaki K., Kakimoto T. (2011). Analysis of cytokinin mutants and regulation of cytokinin metabolic genes reveals important regulatory roles of cytokinins in drought, salt and abscisic acid responses, and abscisic acid biosynthesis. Plant Cell.

[63] Jackson M.L. (1958). Soil Chemical Analysis.

[64] Nakano Y., Asada K. (1981). Hydrogen Peroxide Is Scavenged by Ascorbate-Specific Peroxidase in Spinach Chloroplasts. Plant Cell Physiol..

[65] Aebi H.E. (1983). Catalase. Methods of Enzymatic Analysis.

[66] Kara M., Mishra D. (1976). Catalase, peroxidase, polyphenoloxidase activities during since leaf senescence. Plant Physiol..

[67] Hammerschmidt R., Nuckles E.M., Ku´c J. (1982). Association of enhanced peroxidase activity with induced systemic resistance of cucumber to Colletotrichum lagenarium. Physiol. Plant Pathol..

[68] Davenport S.B., Gallego S.M., Benavides M.P., Tomaro M.L. (2003). Behavior of antioxidant defense system in the adaptive response to salt stress in (Helianthus annuus L.) cell. Plant Growth Reg..

[69] Association of Official Agricultural Chemists (1975). Official Methods of Analysis.

